# Comment on Niemann et al. Outcome Analysis of the Use of Cerament^®^ in Patients with Chronic Osteomyelitis and Corticomedullary Defects. *Diagnostics* 2022, *12*, 1207

**DOI:** 10.3390/diagnostics12102343

**Published:** 2022-09-28

**Authors:** Martin McNally, Jamie Ferguson, Matthew Scarborough, Alex Ramsden, Bridget Atkins

**Affiliations:** 1 The Bone Infection Unit, Nuffield Orthopaedic Centre, Oxford University Hospitals NHS Trust, Windmill Rd., Headington, Oxford OX3 7HE, UK; 2 Department of Infectious Diseases, Oxford University Hospitals NHS Trust, Windmill Rd., Headington, Oxford OX3 7HE, UK; 3 Department of Plastic Surgery, Oxford University Hospitals NHS Trust, Windmill Rd., Headington, Oxford OX3 7HE, UK

We read the recent study by Niemann et al. (2022) [1]. This study contains several incorrect statements and misreports our previous studies and those of other workers. It misquotes references which are not supportive of the text and presents an interpretation of their results which may be harmful to patient care.

The study offers a report on a small retrospective series of 20 patients treated over 5 years (mean 4 patients/year). The authors reported a 50% reoperation rate, mainly due to wound leakage or dehiscence. They compared their results to our better results (6% wound leaks and no wound dehiscence in a prospective series of 100 patients) [2] and stated that this difference was because, “*McNally et al. did not count local problems, such as prolonged wound leakage or dehiscences, as complications*”. This is not correct and may hide the real reason for their high reoperation rate. In our paper, we listed each wound leak with its site, time of onset, duration, treatment and outcome in a section entitled “Complications”. We also summarized these data in a table entitled “Secondary Complications” [2].

Niemann et al. believe that “*…the outcome data presented in the present paper appears to be more realistic as real-world data for most centers*”. This is hard to understand, as they are reporting on a highly selected, non-consecutive, retrospective series of just 20 cases, chosen from the several hundred patients treated in their center over 5 years. There is no information on how these cases were selected for this treatment or why other cases were treated differently. We would contend that our two large, unselected, prospective, consecutive series of 100 and 163 patients represent the real-world of cases seen in many centers [2,3].

The primary outcome measure of this study was “*the frequency of revision surgeries…*”. However, it should be noted that the decision to reoperate is in the hands of the surgeons. Niemann et al. stated that “10 (of their 20 patients) needed at least one revision surgery during the study period due to OM persistence or local wound complications”. They decided to operate on six (30%) of their cases due to a wound leak. These operations occurred between day 4 and day 39 after surgery. It is not possible that wound problems in the first few days are due to the implanted antibiotic material, as it has not started to dissolve to any extent by day 4. It is much more likely that these early reoperations are due to poor wound closure after initial surgery.

It has been widely published that the early leakage of antibiotic materials can be managed conservatively, with excellent results [2,3,4,5,6,7,8]. The decision to reoperate early on 30% of patients was against the advice of the manufacturers of the antibiotic material and the published literature. If these patients did have wound leaks due to the normal dissolution of the material, this could have been safely managed with compression dressings, provided that the patient remained systemically well.

Careful management of the soft tissues is essential in osteomyelitis surgery [2,4,9,10]. Niemann et al. stated that they used a ‘single-stage protocol’ and reference this with two of our papers [2,11]. However, they did not use our single-stage protocol. In that protocol, we described primary wound closure as an essential part of the treatment in every case. Niemann et al. stated that they managed the wounds ‘*preferably with primary wound closure*’ but, unlike our studies, they gave no details on the soft tissue management. This is important because 55% (11/20) of their cases affected the tibia. In our series, over one-fifth of these cases required plastic surgical reconstruction to enable closure. Failure to do this, or choosing to leave the wound open, will undoubtedly increase the need for early revision surgery to achieve wound healing. This condemns the patient to an unplanned two-stage approach.

The authors claimed that they were the first to provide functional scores and PROMS for chronic OM patients. They presented a 50% follow-up rate (10/20) for this, and some of their scores were reported for just 2 or 3 patients. The claim is surprising, as they quote our paper [12], which is a prospective study of 71 chronic OM patients, all assessed with Euro-QoL-5D-3L and EQ-VAS, published in 2020.

Throughout the paper, references are added to imply support for the text, but often the quoted reference has nothing to do with the text. For example, the statement “*Therefore, standardized antibiotic concepts and surgical treatment strategies have been elaborated, which comprise single-stage and two-stage treatment protocols*” is referenced with an editorial [13] which is about surgeon decision-making in regard to infected tibial non-unions and has nothing to do with standardized antibiotic concepts or protocols.

It is important, when presenting new research, to use references and report data from published papers accurately. It is equally important to understand lessons learnt from the new research. It is not reasonable to misquote other papers or misrepresent the content of those papers. In this study, Niemann et al. have ignored the evidence included in previous work, failed to appreciate or report the effect of their management of the soft tissues and exposed their patients to unnecessary secondary surgery.

## Data Availability

No new data was presented in this comment.
